# Supplementary material for: Squeezed light from an oscillator measured at the rate of oscillation
Source: Nat Commun. 2024 May 16;15:4146. doi: 10.1038/s41467-024-47906-0 (PMC11099115; doi:10.1038/s41467-024-47906-0)
Supplement: Supplementary file 1 — Supplementary Information [file 41467_2024_47906_MOESM1_ESM.pdf]

Supplementary Information

Squeezed light from an oscillator measured at the rate of oscillation

Christian Bærentsen,[1] Sergey A. Fedorov,[1,*] Christoffer Østfeldt,[1]
Mikhail V. Balabas,[1] Emil Zeuthen,[1] and Eugene S. Polzik[1,†]
[1]*Niels Bohr Institute, University of Copenhagen, Copenhagen, Denmark*

CONTENTS

A. Experimental setup SI 1

B. The modes of an ensemble of moving atoms interacting with light SI 3
  a. The interaction Hamiltonian SI 3
  b. The Heisenberg-Langevin equations SI 5
  c. Optical damping or anti-damping via dynamical backaction SI 6
  d. The intrinsic dissipation and effective bath temperature for the spin oscillators SI 6
  e. The Heisenberg-Langevin equations for the fast-decaying spin oscillator modes SI 7

C. Homodyne detection and the measurement backaction-imprecision product SI 8

D. The modeling of the experimental data SI 8

E. The sign of the mass SI 10

F. The analytical expressions for the spectra of optical squeezing SI 11
  a. The simplest case—a single oscillator with pure position-measurement interaction SI 11
  b. Optical squeezing in the presence of dynamical backaction SI 12

G. The generation of the collimated tophat beam SI 13

References SI 14

Appendix A: Experimental setup

A detailed schematic of our experimental setup is presented in Fig. SI1a. The probed cesium-133 atoms are located in a channel of a glass chip with $1\,\mathrm{mm}\times 1\,\mathrm{mm}$ cross-section and $40\,\mathrm{mm}$ length. The chip is enclosed in a glass cell, which has a stem attached to it that contains a piece of cesium metal providing a reservoir of atoms. The cell interior is coated with an anti-spin-relaxation paraffin coating to decrease the decoherence due to the collisions of atoms with walls. The cell is heated to $(52 \pm 2)\,^\circ\mathrm{C}$ and placed in a stationary homogeneous magnetic field directed along the x axis, which is created by a pair of rectangular coils parallel to the yz plane. Additional time-dependent magnetic field directed along the y axis can be created using another pair of coils parallel to the xz plane, which has the effect of applying a classical force to the atomic oscillator. The cell and the entire set of coils are enclosed in a multi-layer magnetic shield, including μ-metal layers to eliminate the magnetic field of the Earth and an aluminum layer to protect the spins from external high-frequency magnetic noise.

The atoms interact with two light beams: the probe, which is linearly polarized and propagates along the channel, and the repump, which is circularly polarized and propagates perpendicular to the channel, along the x axis. Both light beams have wavelengths around $852.3\,\mathrm{nm}$, close to the D2 transition from the ground state of Cs (the atomic levels relevant to our experiment are shown in Fig. SI1b). The ground state of Cs is split into two hyperfine levels, with the magnetic momentum numbers $F = 3$ and $F = 4$, and each hyperfine level is further split into $(2F + 1)$ magnetic sublevels. The repump beam is produced by a diode laser and has the power in the range of $8 - 10\,\mathrm{mW}$. It is blue-detuned by $80\,\mathrm{MHz}$ from the $F = 3 \to F' = 2$ transition of the D2 line, and resonant with all transitions

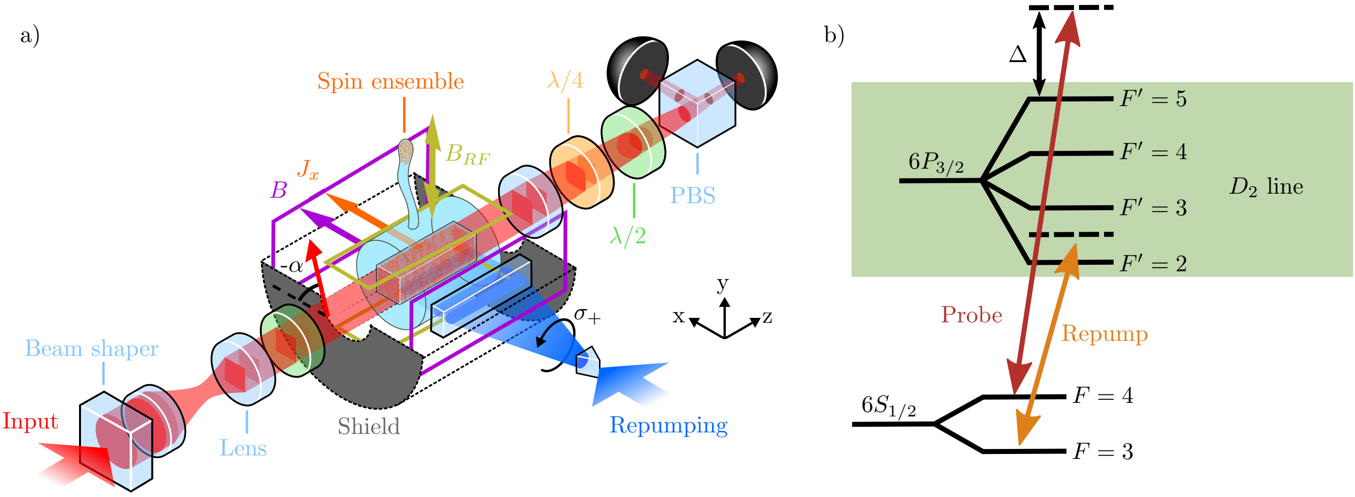

FIG. SI1. a) Experimental setup. A linearly polarized light probe is spatially shaped as a square-tophat beam. The probe interacts with an optically polarized ensemble of Cesium atoms located in a glass chip. The macroscopic atomic polarization J_x is oriented along the magnetic field B. The optical probe and the atomic ensemble interact via Faraday interaction in the dispersive regime. The output probe light is detected using a polarization self-homodyning setup. PBS: Polarizing beamsplitter. $\lambda/2$: Half wave plate. $\lambda/4$: Quarter wave plate. Beam shaper: Gaussian-to-tophat beam-shaping lens. b) Optical level scheme for probing and repumping.

$F = 3 \rightarrow F' = 2, 3, 4$ within the Doppler linewidth, where the primes denote electronically excited states. The cross-section of the chip channel containing atoms is chosen to be square to avoid lensing of the repump beam. In order to uniformly illuminate the elongated channel, the repump beam is shaped by a combination of a Powell lens and a cylindrical collimating lens. The repump transfers all atoms to $F = 4$ level, and simultaneously creates macroscopic spin orientation in the ensemble because of its circular polarization. The chirality of the polarization, σ_+ or σ_-, determines the sign of the mass of the oscillator [1]. Our experiments are done with a negative-mass oscillator, but the results, including the observed levels of squeezing, are largely independent of the sign of the mass (see Sec. E). The probe beam is blue-detuned by $0.7 - 7\,\text{GHz}$ from $F = 4 \rightarrow F' = 5$ transition; it is produced by a Ti:Sa laser and has the power up to $13\,\text{mW}$. The probe interacts with the ensemble in the dispersive regime, but the residual spontaneous scattering of its photons does contribute to the spin decoherence. The linear polarization of the probe is set along the y axis to maximize the optical damping by the dynamical backaction (which nevertheless remains small, see Sec. Bc), and simultaneously decouple the spin from the classical intensity fluctuations. The small amount of optical damping in our experiments improves the maximum observed level of squeezing (see Sec. F). The decoherence rate due to the spontaneous scattering is proportional to the probe power, and is the primary limitation for the achievable quantum cooperativity in our work.

The distribution of the atoms among the magnetic sublevels is determined by the interplay of the spontaneous scattering processes due to the probe and the repump beam, and is independent of the probe power and detuning within our range of parameters. The mean polarization of the spin is

$$\frac{1}{NF}\langle \hat{J}_x \rangle = \frac{1}{FN}\sum_{m=-F}^{F} m\, N_m = 0.78 \pm 0.04, \qquad \text{(SI A.1)}$$

as characterized using the Magneto-Optical Resonance Signal method [2]. The distribution of the atomic populations N_m over the magnetic sublevels is to a very good degree exponential, $N_m \propto \exp(\beta m)$, with $\beta \approx 0.75$, from which the populations of the individual m-states can be inferred.

After the interaction with the atomic ensemble, the relevant quadratures of the probe beam are detected using polarization homodyning. The quadrature angle is selected using a combination of a quarter waveplate and a half waveplate. A key advantage of the polarization homodyning method is the perfect spatial overlap between the detected modes of light and the local oscillator. The electronic noise floor of the photodetector is typically about $30\,\text{dB}$ below the shot noise level and hence is negligible.

The maximum narrowband squeezing of light observed in the regime when $\Gamma \ll |\Omega_S|$ is approximately independent of the Larmor frequency within the range of Larmor frequencies between $0.8\,\text{MHz}$ and $5\,\text{MHz}$. At low frequencies,

the limitation is due to classical noises acting on the spins, and at high frequencies due to the inhomogeneity of the magnetic field, which could be straightforwardly improved.

In order to minimize the coupling to the fast-decaying modes of the spin ensemble (see Sec. B), the probe beam is shaped into a square tophat beam using a high-transmission beam shaping lens (Topag GTH-3.6-1.75FA), and an additional system of regular spherical lenses described in Sec. G. The resulting beam has a supergaussian intensity cross section $I(x, y) \propto \exp(-2(x/w_x)^{2n} - 2(y/w_y)^{2n})$ with $n \approx 3.2$ and $2w_x \approx 2w_y \approx 0.84\,\mathrm{mm}$, which change negligibly in the z direction over the length of the cell channel. The on-resonance extraneous thermal noise in the slow-measurement regime was experimentally found to be lower by a factor of 3.6 for the tophat beam probe compared to the Gaussian beam probe with the maximum width allowed by the cell channel. The transmission of the probe beam through the cell reaches 96.8%, limited by the reflection and scattering of light upon hitting the cell windows, with the loss of light due to the clipping of the beam being negligible. In order to infer the generated level of squeezing from detected, we assume that the transmission loss is equally contributed by the input and the output windows.

Appendix B: The modes of an ensemble of moving atoms interacting with light

a. The interaction Hamiltonian

In this section, we describe N moving atoms interacting with the probe light field, and derive input-output relations for the optical quadratures in terms of two types of collective spin oscillator modes: usual Larmor precession modes, and modes scrambled by the atomic motion. The main assumptions that underly our treatment of the atom-light interaction are the following:

1. The intensities of all laser drives are far below the saturation intensities of the atomic transitions.
2. The number of atoms in the ensemble is macroscopic, meaning that the relative changes in the numbers of atoms in every particular magnetic sub-state due to the vacuum fluctuations of the probe light are negligible.
3. The density of atoms is low enough for the photons once incoherently scattered within the ensemble to leave the ensemble without being scattered again.
4. The coherent amplitude of the probe light is large compared to its fluctuations, and does not change as the light propagates through the atomic ensemble.

Under these assumptions, we let the individual atoms interact with the light field with the strengths $g_k(t)$ (where $k = 1, .., N$ is the integer index that labels the atoms) that is proportional to the intensity of the light field at their instantaneous positions. The interaction strengths randomly change in time as atoms move inside the cell. The motions of different atoms are assumed to have the same statistical properties and be uncorrelated between each other. The statistics of motion are characterized by decomposing the couplings into their mean value, $\bar{g}$, and deviations, $\Delta g_k(t)$,

$$g_k(t) = \bar{g} + \Delta g_k(t), \tag{SI B.1}$$

and specifying the motional correlation function, $R(\tau)$,

$$\frac{\langle \Delta g_k(t_1)\, \Delta g_l(t_2)\rangle_c}{\langle \Delta g(t)^2\rangle_c} = \delta_{kl} R(t_1 - t_2), \tag{SI B.2}$$

where δ_{kl} is the Kronecker symbol and $\langle\cdot\rangle_c$ denotes motional averaging (following the notation of Ref. [3], to separate from the quantum averaging $\langle\cdot\rangle$). The normalization factor, $\langle \Delta g(t)^2\rangle_c$, is the mean squared deviation among the individual atom-light couplings. According to the ergodic hypothesis, the result of the averaging is the same regardless of whether it is done over the time or the realizations of the ensemble.

The dispersive interaction between the light and the $k-$th atom in the ensemble is described by the Hamiltonian [4, 5]

$$\hat{H}^{(k)}_{\text{int}} = \hbar\, g_k(t) \left[a_0 \hat{I} + a_1 \hat{S}_z \hat{j}^{(k)}_z + a_2 \left(\hat{I}\, \hat{j}^{(k)}_z \hat{j}^{(k)}_z - 2\hat{S}_x \left(\hat{j}^{(k)}_x \hat{j}^{(k)}_x - \hat{j}^{(k)}_y \hat{j}^{(k)}_y\right) - 2\hat{S}_y \left(\hat{j}^{(k)}_x \hat{j}^{(k)}_y + \hat{j}^{(k)}_y \hat{j}^{(k)}_x\right)\right)\right], \tag{SI B.3}$$

where $\hat{S}_{x,y,z}$ are the Stokes parameters of the input light [4], $\hat{I}$ is the intensity of the input light, and the parameters $a_{0,1,2}$ are functions of the level structure and the laser detuning from the optical transition [6]. After linearization

assuming a strong coherent y-polarized light probe with the mean amplitude $\bar{a}$, the Hamiltonian is expressed as

$$\hat{H}^{(k)}_{\text{int}} = \hat{H}^{(k)}_{\text{Stark}} - \hbar \frac{\bar{a} g_k(t)}{\sqrt{2}} \left[a_1 \hat{j}^{(k)}_z \hat{X}_{\text{L}} - 2a_2 \left(\hat{j}^{(k)}_x \hat{j}^{(k)}_y + \hat{j}^{(k)}_y \hat{j}^{(k)}_x \right) \hat{P}_{\text{L}} \right], \tag{SI B.4}$$

where the Stark Hamiltonian $\hat{H}^{(k)}_{\text{Stark}} = \hbar g_k(t) \left[a_0 + a_2 \left(\hat{j}^{(k)}_x \hat{j}^{(k)}_x - \hat{j}^{(k)}_y \hat{j}^{(k)}_y + \hat{j}^{(k)}_z \hat{j}^{(k)}_z \right) \right] \hat{I}$ describes the energy shifts due to the dynamic Stark effect, and $\hat{X}_{\text{L}}$ and $\hat{P}_{\text{L}}$ are the polarization quadratures of the light field normalized such that they satisfy the commutation relation

$$[\hat{X}_{\text{L}}(t_1), \hat{P}_{\text{L}}(t_2)] = (i/2)\delta(t_1 - t_2). \tag{SI B.5}$$

The spin components of individual atoms $\hat{j}^{(k)}_{x,y,z}$ can be expressed in terms of the jump operators $\hat{\sigma}^{(k)}_{n,m}$ between the ground state sublevels,

$$\hat{\sigma}^{(k)}_{n,m} = |n\rangle_k \langle m|_k, \tag{SI B.6}$$

where $m, n = -F, ..., F$ is the projection of the angular momentum on the x axis (which coincides with the direction of the magnetic field), and F is the total angular momentum quantum number of the ground state level. In this notation,

$$H^{(k)}_{\text{int}} = \hat{H}^{(k)}_{\text{Stark}} + \hbar \frac{\bar{a} g_k(t)}{2\sqrt{2}} \sum_{m=-F}^{F-1} C_m \left(i a_1 \left(\hat{\sigma}^{(k)}_{m+1,m} - \hat{\sigma}^{(k)}_{m,m+1} \right) \hat{X}_{\text{L}} + 2(2m+1) a_2 \left(\hat{\sigma}^{(k)}_{m+1,m} + \hat{\sigma}^{(k)}_{m,m+1} \right) \hat{P}_{\text{L}} \right), \tag{SI B.7}$$

where $\hat{H}^{(k)}_{\text{Stark}} = \hbar \sum_m g_k(t) \left(a_0 + a_2 m^2 \right) \hat{I}\, \hat{\sigma}^{(k)}_{m,m}$ is the Stark energy, and $C_m = \sqrt{F(F+1) - m(m+1)}$ are Clebsch–Gordan coefficients. When transiting from Eq. (SI B.4) to Eq. (SI B.7) we neglected the terms involving second-order coherences that only couple to $\hat{I}$ and are negligibly small in our case.

The individual atomic spins are precessing in a homogeneous magnetic field directed along the x axis. Taking the zero of the energy scale to be the ground state energy of free atoms, the Hamiltonian of the precession is expressed as

$$\hat{H}^{(k)}_{\text{S}} = \sum_{m=-F}^{F} E_{\text{Zeem},m}\, \hat{\sigma}^{(k)}_{m,m}, \tag{SI B.8}$$

where $E_{\text{Zeem},m}$ are the Zeeman energies of the magnetic sublevels that include contributions linear and quadratic in m. The total Hamiltonian of all atoms,

$$\hat{H} = \sum_{k=1}^{N} \left(\hat{H}^{(k)}_{\text{S}} + \hat{H}^{(k)}_{\text{int}} \right), \tag{SI B.9}$$

can be expressed using collective operators: the total numbers of atoms in the magnetic sublevels, denoted by $\hat{N}_m$, and two sets of coherences between neighboring m levels, denoted by $\hat{\Sigma}_m$ and $\hat{\Sigma}'_m$. The operators are defined as

$$\hat{N}_m = \sum_{k=1}^{N} \hat{\sigma}^{(k)}_{m,m}, \qquad \hat{\Sigma}_m = \sum_{k=1}^{N} \hat{\sigma}^{(k)}_{m+1,m}, \qquad \hat{\Sigma}'_m = \frac{1}{\sqrt{\langle \Delta g^2 \rangle_c}} \sum_{k=1}^{N} \Delta g_k(t)\, \hat{\sigma}^{(k)}_{m+1,m}, \tag{SI B.10}$$

where $m = -F, ..., F-1$ for the Σ operators and $m = -F, ..., F$ for the N operators. The expression for the Hamiltonian, neglecting a small contribution due to the inhomogeneity of the Stark shift, is

$$\begin{aligned} \hat{H} = \sum_{m=-F}^{F} E_m \hat{N}_m + \hbar \sum_{m=-F}^{F-1} \frac{\bar{g} \bar{a} a_1}{2\sqrt{2}} C_m \left(i \left(\hat{\Sigma}_m - \hat{\Sigma}^\dagger_m \right) \hat{X}_{\text{L}} + \zeta_m \left(\hat{\Sigma}_m + \hat{\Sigma}^\dagger_m \right) \hat{P}_{\text{L}} \right) \\ + \hbar \sum_{m=-F}^{F-1} \frac{\sqrt{\langle \Delta g^2 \rangle_c}\, \bar{a} a_1}{2\sqrt{2}} C_m \left(i \left(\hat{\Sigma}'_m - \hat{\Sigma}'^\dagger_m \right) \hat{X}_{\text{L}} + \zeta_m \left(\hat{\Sigma}'_m + \hat{\Sigma}'^\dagger_m \right) \hat{P}_{\text{L}} \right), \end{aligned} \tag{SI B.11}$$

where $\zeta_m = 2(2m+1)a_2/a_1$, and $E_m = E_{\text{Zeem},m} + E_{\text{Stark},m}$ is the sum of the Zeeman and the Stark energies. In the limit of a large number of atoms in the ensemble, the two sets of $\hat{\Sigma}_m$ operators are independent and have constant commutators,

$$\left[\hat{\Sigma}_n, \hat{\Sigma}_m^\dagger\right] = \delta_{nm}\left(\hat{N}_{m+1} - \hat{N}_m\right) \underset{N\gg 1}{\longrightarrow} \delta_{nm}\left(N_{m+1} - N_m\right), \tag{SI B.12}$$

$$\left[\hat{\Sigma}_n, \hat{\Sigma}_m'^\dagger\right] = \delta_{nm}\sum_k \frac{\Delta g_k(t)}{\sqrt{\langle \Delta g^2\rangle_c}}\left(\hat{\sigma}^{(k)}_{m+1,m+1} - \hat{\sigma}^{(k)}_{m,m}\right) \underset{N\gg 1}{\longrightarrow} 0, \tag{SI B.13}$$

$$\left[\hat{\Sigma}_n', \hat{\Sigma}_m'^\dagger\right] = \delta_{nm}\sum_j \frac{\Delta g_j(t)^2}{\langle \Delta g^2\rangle_c}\left(\hat{\sigma}^{(j)}_{m+1,m+1} - \hat{\sigma}^{(j)}_{m,m}\right) \underset{N\gg 1}{\longrightarrow} \delta_{nm}\left(N_{m+1} - N_m\right), \tag{SI B.14}$$

where $m, n = -F, ..., F-1$, and $N_m = \langle \hat{N}\rangle$ are the average macroscopic populations of the magnetic sublevels. By normalizing the Σ operators to satisfy the canonic commutation relations, we can introduce two sets of bosonic modes, $\hat{b}_m$ and $\hat{b}_m'$, that appear in the main text,

$$\hat{b}_m = \hat{\Sigma}_m/\sqrt{\Delta N_m}, \qquad \hat{b}_m' = \hat{\Sigma}_m'/\sqrt{\Delta N_m}, \tag{SI B.15}$$

where $\Delta N_m = N_{m+1} - N_m$. The modes described by $\hat{b}_m$ are those usually identified with the Larmor precession of the spin ensemble as a whole. They experience coupling to the probe light that is averaged over the atomic trajectories [7], and their coherence time is high, limited by the reorientation of individual spins due to the collisions with the walls and between each other, and by the spontaneous scattering of probe photons. The modes described by $\hat{b}_m'$ experience additional damping and decoherence due to the atoms flying in and out of the probe beam. We refer to them as the fast-decaying modes. Introducing the quadratures of the spin oscillators,

$$\hat{X}_m \equiv \frac{1}{i\sqrt{2}}\left(\hat{b}_m - \hat{b}_m^\dagger\right), \quad \hat{P}_m \equiv -\frac{1}{\sqrt{2}}\left(\hat{b}_m + \hat{b}_m^\dagger\right), \quad \hat{X}_m' \equiv \frac{1}{i\sqrt{2}}\left(\hat{b}_m' - \hat{b}_m'^\dagger\right), \quad \hat{P}_m' \equiv -\frac{1}{\sqrt{2}}\left(\hat{b}_m' + \hat{b}_m'^\dagger\right), \tag{SI B.16}$$

which satisfy $[\hat{X}_m, \hat{P}_m] = i$ and $[\hat{X}_m', \hat{P}_m'] = i$, and using the fact that, in the Holstein-Primakoff approximation, the numbers of atoms in the $m-$th levels satisfy

$$\hat{N}_m \approx N_m + \frac{1}{2}\left(\hat{b}_m^\dagger\hat{b}_m + \hat{b}_m'^\dagger\hat{b}_m' - \hat{b}_{m-1}^\dagger\hat{b}_{m-1} - \hat{b}_{m-1}'^\dagger\hat{b}_{m-1}' + \text{h.c.}\right), \tag{SI B.17}$$

the total Hamiltonian in Eq. (SI B.11) is expressed as

$$\hat{H} = \hbar\sum_{m=-F}^{F-1}\left[\frac{\Omega_m}{2}\left(\hat{X}_m^2 + \hat{P}_m^2\right) + \frac{\Omega_m}{2}\left(\hat{X}_m'^2 + \hat{P}_m'^2\right) - 2\sqrt{\Gamma_m}\left(\hat{X}_m\hat{X}_\text{L} + \zeta_m\hat{P}_m\hat{P}_\text{L}\right) - 2\sqrt{\Gamma_m'}\left(\hat{X}_m'\hat{X}_\text{L} + \zeta_m\hat{P}_m'\hat{P}_\text{L}\right)\right], \tag{SI B.18}$$

which is a Hamiltonian of $4F$ oscillators linearly coupled to a propagating field. The frequencies Ω_m are determined by the energy splittings between different magnetic sublevels due to the Zeeman and Stark effects,

$$\hbar\Omega_m = E_{\text{Zeem},m} - E_{\text{Zeem},m+1} - \hbar\bar{g}a_2I(2m+1), \tag{SI B.19}$$

and the measurement rates for the slow- and the fast-decaying modes are identified as

$$\Gamma_m = \bar{g}^2(\bar{a}a_1\,C_m)^2\Delta N_m/16, \qquad \Gamma_m' = \langle \Delta g^2\rangle_c\,(\bar{a}a_1\,C_m)^2\Delta N_m/16. \tag{SI B.20}$$

b. The Heisenberg-Langevin equations

The Hamiltonian in Eq. (SI B.18) describes the linear interaction of the localized collective atomic spin oscillators with the propagating optical field. The evolution of the variables of the system can be found by solving the Heisenberg-Langevin equations for the spin combined with the input-output relations for the light field. We derive these equations

by using the well-known procedure outlined in Ref. [8, 9], arriving on the following input-output relations

$$\hat{X}_{\mathrm{L}}^{\mathrm{out}}(t) = \hat{X}_{\mathrm{L}}^{\mathrm{in}}(t) - \sum_{m=-F}^{F-1} \zeta_m \left(\sqrt{\Gamma_m}\hat{P}_m(t) + \sqrt{\Gamma'_m}\hat{P}'_m(t)\right), \tag{SI B.21}$$

$$\hat{P}_{\mathrm{L}}^{\mathrm{out}}(t) = \hat{P}_{\mathrm{L}}^{\mathrm{in}}(t) + \sum_{m=-F}^{F-1} \left(\sqrt{\Gamma_m}\hat{X}_m(t) + \sqrt{\Gamma'_m}\hat{X}'_m(t)\right), \tag{SI B.22}$$

and the Heisenberg equations of motion (for the slow-decaying modes only, for now),

$$\frac{d}{dt}\hat{b}_m(t) = -i\left[\hat{b}_m, \hat{H}\right], \tag{SI B.23}$$

expressed in terms of the quadratures as

$$\frac{d}{dt}\hat{X}_m(t) = \Omega_m\hat{P}_m(t) - \sum_{n=-F}^{F-1} \zeta_m\sqrt{\Gamma_m}\left(\sqrt{\Gamma_n}\hat{X}_n(t) + \sqrt{\Gamma'_n}\hat{X}'_n(t)\right) - 2\zeta_m\sqrt{\Gamma_m}\hat{P}_{\mathrm{L}}^{\mathrm{in}}(t), \tag{SI B.24}$$

$$\frac{d}{dt}\hat{P}_m(t) = -\Omega_m\hat{X}_m(t) - \sum_{n=-F}^{F-1} \zeta_n\sqrt{\Gamma_m}\left(\sqrt{\Gamma_n}\hat{P}_n(t) + \sqrt{\Gamma'_n}\hat{P}'_n(t)\right) + 2\sqrt{\Gamma_m}\hat{X}_{\mathrm{L}}^{\mathrm{in}}(t). \tag{SI B.25}$$

Above, we assumed the Markov approximation for the optical field, already implicitly introduced before when stating that the commutator of $\hat{X}_{\mathrm{L}}$ and $\hat{P}_{\mathrm{L}}$ is a delta-function in Eq. (SI B.5).

c. *Optical damping or anti-damping via dynamical backaction*

Eq. (SI B.24-SI B.25) describing the motion of the spin oscillators contain two kinds of terms arising due to the interaction with the optical field. The first kind are the terms involving the input variables of light, $\hat{X}_{\mathrm{L}}^{\mathrm{in}}(t)$ and $\hat{P}_{\mathrm{L}}^{\mathrm{in}}(t)$. They are, in the quasi-classical language, responsible for driving the spin motion by the quantum measurement backaction. The second kind are the terms coupling the spin variables between each other—these terms are collected under the sums over n. For each individual oscillator mode m they prescribe damping at the rate $\gamma_{\mathrm{DBA},m} = 2\zeta_m\Gamma_m$ (which can be negative, if $\zeta_m < 0$), and for each pair of the oscillator modes n and k they prescribe coupling at the rates $\sqrt{\gamma_{\mathrm{DBA},k}\gamma_{\mathrm{DBA},n}}$. The combined effect of the terms of the second kind is referred to as dynamical backaction.

Dynamical backaction is a term originally introduced to describe the modification of the oscillator damping rate and resonance frequency due to its coupling to a measuring device, arising in the context of continuous force measurements with mechanical probes [10]. Later, this term was widely adopted to describe the coherent modification of the probed system dynamics by light in optomechanics, magnonomechanics, and levitated nanoparticles.

In our model of the light-spin interaction, which assumes Markovian optical reservoir and direct coupling between the light and the spin (not, for example, via an optical cavity), the dynamical backaction can only amplify or dampen the oscillator motion, and couple different modes, but not shift their resonance frequencies. This is another way of saying that $\gamma_{\mathrm{DBA},m}$ are always real. The tensor interaction coefficients ζ_m that determine the strengths of the dynamical backaction effects have absolute values between 0.01 and 0.18 in our experiments. They scale with the optical detuning approximately as $1/\Delta$, making the dynamical backaction effects the strongest at small optical detunings, were we also can achieve the largest measurement rates Γ.

It should also be noted that the optical damping via dynamical backaction is a different effect than the increase of the intrinsic damping rate of the spin due to the spontaneous scattering (which is sometimes referred to as "power broadening", and also present in our experiments). While both dynamical backaction damping and "power broadening" are proportional to the probe power, at a fixed probe power they have different scalings with the optical depth along the probe propagation direction—dynamical backaction damping is proportional to the optical depth, and "power broadening" is independent of the optical depth.

d. *The intrinsic dissipation and effective bath temperature for the spin oscillators*

In addition to the coupling to the light field, the spin oscillators experience intrinsic dissipation, primarily due to the spontaneous scattering of probe and repump photons, and to a lesser extent due to the collisions with walls and

between each other. The intrinsic dissipation is added to Eq. (SI B.24-SI B.25) using the same quantum Langevin approach [8] that was used for the optical field. It was outlined for the case of atomic ensembles, e.g., in Ref. [5]. The intrinsic dissipation modifies Eq. (SI B.23) as

$$\frac{d}{dt}\hat{b}_m(t) = -i\left[\hat{b}_m, \hat{H}\right] - \frac{\gamma_0}{2}\hat{b}_m(t) + \sqrt{\gamma_0}\hat{\mathcal{F}}_m(t), \tag{SI B.26}$$

where $\langle\hat{\mathcal{F}}_m^\dagger(t_1)\hat{\mathcal{F}}_m(t_2)\rangle = n_{\text{th}}\delta(t_1-t_2)$ and $\langle\hat{\mathcal{F}}_m(t_1)\hat{\mathcal{F}}_m^\dagger(t_2)\rangle = (n_{\text{th}}+1)\delta(t_1-t_2)$.

The temperatures of the effective thermal baths of the oscillator modes, n_{th}, can be determined from the equilibrium numbers of excitation in the modes in the absence of probing,

$$n_{\text{th}} = \langle\hat{b}_m^\dagger\hat{b}_m\rangle = N_m/\Delta N_m. \tag{SI B.27}$$

This formula follows directly from the definition of $\hat{b}_m$ in Eq. (SI B.15) under the assumption that the scattering processes that determine the equilibrium populations N_m affect all atoms independently.

The thermal occupancies of the intrinsic baths damping the oscillators with different indices m do not need to be identical in principle, because they depend in a complex way on the Clebsch-Gordan coefficients and the details of the atomic collisions. Experimentally, however, by characterizing the steady-state distribution of the population of atoms over the magnetic sublevels using the Magneto-Optical Resonance Signal [2, 11], we found that $N_m/\Delta N_m$ are the same for all m within our measurement precision, and hence the temperature of the effective oscillator bath is independent of m. This means that the steady-state distribution of the population of atoms N_m in our experiments is approximately exponential,

$$\frac{N_{m+1}}{N_m} \approx \exp\left(\frac{k_{\text{B}}T}{\hbar\Omega_L}\right), \tag{SI B.28}$$

with the same effective temperature T for all m. For this reason, we do not indicate the m index when providing the value for n_{th} in the main text.

e. The Heisenberg-Langevin equations for the fast-decaying spin oscillator modes

The Heisenberg equations of motion describing the evolution of the modes from the fast-decaying family are identical to Eq. (SI B.24-SI B.25), except that they include additional terms due to the explicit time dependence of their operators. These terms are more convenient to present for the annihilation operators than for the quadratures, they are given by

$$\frac{d}{dt}\hat{b}'_m(t) = -i\left[\hat{b}'_m, \hat{H}\right] + \frac{1}{\sqrt{\Delta N_m\,\langle\Delta g^2\rangle_c}}\sum_{k=1}^{N}\left(\frac{d}{dt}\Delta g_k(t)\right)\hat{\sigma}^{(k)}_{m+1,m}, \tag{SI B.29}$$

where $-i[\hat{b}'_m, \hat{H}]$ contributes the terms due to the coherent evolution and the coupling to the light field that are completely analogous to those present in Eq. (SI B.24-SI B.25). The added terms give rise to both extra dissipation and fluctuations. If the motional correlation function is exponential, $\langle\Delta g_k(t_1)\,\Delta g_k(t_2)\rangle \propto e^{-\gamma_b|t_1-t_2|/2}$, as it was suggested in [7], the stochastic evolution of $\Delta g_k(t)$ can be modeled by the Ornstein–Uhlenbeck process,

$$\frac{d}{dt}\Delta g_k(t) = -\frac{\gamma_b}{2}\Delta g_k(t) + \sqrt{\gamma_b}f_k(t), \tag{SI B.30}$$

where $\langle f_k(t_1)f_k(t_2)\rangle_c = \langle\Delta g^2\rangle_c\,\delta(t_1-t_2)$. In this case, the extra terms in the Heisenberg-Langevin equations for $\hat{b}'$ can be re-expressed as

$$\frac{d}{dt}\hat{b}'_m(t) = -i\left[\hat{b}'_m, \hat{H}\right] - \frac{\gamma_b}{2}\hat{b}'_m(t) + \sqrt{\gamma_b}\hat{\mathcal{F}}'_{m,b}(t), \tag{SI B.31}$$

where $\langle\hat{\mathcal{F}}'^\dagger_{m,b}(t_1)\hat{\mathcal{F}}'_{m,b}(t_2)\rangle = n_{\text{th}}\delta(t_1-t_2)$ and $n_{\text{th}} = N_m/\Delta N_m$ is the thermal occupancy of the bath. While the atomic motion increases the decoherence rate, the thermal bath occupancies for the fast- and slow-decaying modes are the same.

Appendix C: Homodyne detection and the measurement backaction-imprecision product

After interaction with the atomic ensemble, the two conjugated quadratures of the probe light, $\hat{X}_{\rm L}^{\rm out}$ and $\hat{P}_{\rm L}^{\rm out}$, as well as any intermediate quadrature $\hat{Q}_{\rm L}^{\phi}$,

$$\hat{Q}_{\rm L}^{\phi}(t) = \sin(\phi)\hat{X}_{\rm L}^{\rm out}(t) + \cos(\phi)\hat{P}_{\rm L}^{\rm out}(t), \tag{SI C.1}$$

can be detected by balanced polarization homodyning by passing the output light through a combination of a half and a quarter waveplates. The rotation angles of the waveplates allow setting the detection angle ϕ. The power spectral density (PSD) of the photocurrent signal is given by

$$S_\phi[\Omega] = \frac{1}{4}(1-\eta) + \eta \int_{-\infty}^{\infty} e^{i\Omega\tau} \left\langle \hat{Q}_{\rm L}^{\phi}(t+\tau)\, \hat{Q}_{\rm L}^{\phi}(t) \right\rangle d\tau, \tag{SI C.2}$$

where η is the detection efficiency. We use two-sided spectral densities, which means that the spectrum of a zero-mean random variable, call it ξ, is defined for positive and negative frequencies, and is related to the variance of ξ as

$$\int_{-\infty}^{\infty} S_\xi[\Omega] \frac{d\omega}{2\pi} = \left\langle \xi^2 \right\rangle. \tag{SI C.3}$$

When the optical field is in the vacuum state, its correlation is given by $\langle \hat{Q}_{\rm L}^{\phi}(t+\tau)\, \hat{Q}_{\rm L}^{\phi}(t)\rangle = (1/4)\delta(\tau)$, and therefore $S_\phi[\Omega] = 1/4$; this value is the shot noise level. The observation $S_\phi[\Omega] < 1/4$ means that some of the Fourier-domain modes of light are in squeezed states.

The spectral density of the photocurrent when the homodyne is tuned to detect the P quadrature is given by

$$S_\phi[\Omega] = \frac{1}{4} + \eta\Gamma\, S_{X_S X_S}[\Omega] + \eta\, S_{PP,\rm ext}[\Omega], \tag{SI C.4}$$

where $S_{X_S X_S}[\Omega]$ is the spectrum of the total spin motion, and $S_{PP,\rm ext}[\Omega]$ is the extraneous noise. In the slow-measurement regime when $\Gamma \ll |\Omega_S|$, $S_{PP,\rm ext}$ comes from the thermal noise of fast-decaying modes (see Sec. D), and in the fast-measurement regime when $\Gamma \sim |\Omega_S|$, $S_{PP,\rm ext} = 0$. There is no detectable extraneous noise in the X quadrature of light in our experiments. The spectrum of the imprecision noise for measurements on the P quadrature is given by

$$S_{\rm imp}[\Omega] = \frac{1/4 + S_{PP,\rm ext}[\Omega]}{\eta\Gamma}, \tag{SI C.5}$$

The spectrum of the backaction noise is given by $S_{\rm BA}[\Omega] = \hbar^2\left(\Gamma(1+\zeta^2) + \gamma_{\rm sc}\right)$, where $\gamma_{\rm sc}$ is the decoherence rate of the oscillator due to spontaneous scattering, which is proportional to the probe power. We conservatively estimate $\gamma_{\rm sc}/\Gamma$ as $1/\mathcal{C}_{\rm q}$ (as if all the decoherence of spin oscillators comes from spontaneous scattering). Overall, the backaction-imprecision product in terms of the two-sided spectral densities is found as

$$\sqrt{S_{\rm imp}\, S_{\rm BA}} = (\hbar/2)\sqrt{\frac{1}{\eta}\left(1 + \frac{S_{PP,\rm ext}}{\rm SN}\right)\left(1 + \zeta^2 + \frac{1}{\mathcal{C}_{\rm q}}\right)}. \tag{SI C.6}$$

where SN $= 1/4$ is the shot noise level. This expression exposes how various imperfections of the measurements, including the finite detection efficiency, the extraneous noise, the "heating" due to spontaneous scattering, and the dynamical backaction, elevate the backaction-imprecision product above the quantum limit of $\hbar/2$ in our experiments.

Appendix D: The modeling of the experimental data

The model we use to fit the experimental data in Fig. 2 and 3 of the main text is based on the empirical assumption that the complex dynamics of the 16 spin oscillator modes introduced in Sec. B can be explained as arising from only two or three "bright" interacting modes with some effective parameters. This assumption is supported by the good agreement that we get between the model and the experimental data using such an approach.

To process the experimental data, we model the homodyne spectrum as arising from the dynamics of several oscillator modes coupled to the probe field, using the input-output relations that are expressed analogously to Eqs.(SI B.21) and (SI B.22),

$$\hat{X}_{\rm L}^{\rm out}(t) = \hat{X}_{\rm L}^{\rm in}(t) - \sum_{i=1}^{n_{\rm modes}} \zeta_i \sqrt{\Gamma_i} \hat{P}_i(t), \qquad \hat{P}_{\rm L}^{\rm out}(t) = \hat{P}_{\rm L}^{\rm in}(t) + \sum_{i=1}^{n_{\rm modes}} \sqrt{\Gamma_i} \hat{X}_i(t). \tag{SI D.1}$$

and the Heisenberg equations of motion analogous to Eqs.(SI B.24) and (SI B.25),

$$\frac{d}{dt}\hat{X}_i(t) = \Omega_i \hat{P}_i(t) - \frac{\gamma_{0,i}}{2}\hat{X}_i(t) - \sum_{j=1}^{n_{\rm modes}} \zeta_i \sqrt{\Gamma_i \Gamma_j} \hat{X}_j(t) - 2\zeta_i \sqrt{\Gamma_i} \hat{P}_{\rm L}^{\rm in}(t) + \hat{F}_i^X(t), \tag{SI D.2}$$

$$\frac{d}{dt}\hat{P}_i(t) = -\Omega_i \hat{X}_i(t) - \frac{\gamma_{0,i}}{2}\hat{P}_i(t) - \sum_{j=1}^{n_{\rm modes}} \zeta_j \sqrt{\Gamma_i \Gamma_j} \hat{P}_j(t) + 2\sqrt{\Gamma_i} \hat{X}_{\rm L}^{\rm in}(t) + \hat{F}_i^P(t). \tag{SI D.3}$$

The index i counts the modes of the model, corresponding to the hybridized resonances we observe in the experimental spectra. The model accounts for the intrinsic dissipation of the modes characterized by the damping rates $\gamma_{0,i}$, and thermal forces $\hat{F}_i^{X,P}(t)$ via the quantum Langevin approach. The correlators of the thermal forces are

$$\left\langle \hat{F}_i^X(t_1)\hat{F}_j^X(t_2) \right\rangle = \left\langle \hat{F}_i^P(t_1)\hat{F}_j^P(t_2) \right\rangle = \delta_{ij}\gamma_{0,i}(n_{\rm th}+1/2)\delta(t_1-t_2), \qquad \left\langle \hat{F}_i^X(t_1)\hat{F}_j^P(t_2) + \hat{F}_j^P(t_2)\hat{F}_i^X(t_1) \right\rangle = 0. \tag{SI D.4}$$

The intrinsic dissipation in our experiments is dominated by spin depolarization due to the atomic collisions and spontaneous scattering of probe photons, which is why we assume that it symmetrically affects X and P, and that the thermal noises are delta-correlated [6]. The thermal occupancy of the intrinsic bath is $n_{\rm th} = 0.9 \pm 0.1$, as extracted from the equilibrium macroscopic population distribution of atoms over the magnetic sublevels.

The fast-decaying modes are treated as one, because their frequency splitting is much smaller than their decoherence rates. This mode is accounted differently for different detunings of the optical probe. At large detunings, the measurement rate for the fast-decaying mode also is much smaller than its decoherence rate, and the dynamic backaction is negligible. In this case, it contributes incoherent thermal noise to the measurement of slow-decaying modes. The spectrum of this noise in the $\hat{P}_{\rm L}$ quadrature of the output light is given by

$$S_{PP,\rm ext}[\Omega] = \Gamma' \int_{-\infty}^{\infty} e^{i(\Omega-\Omega_S)\tau} \frac{\langle \Delta g(t+\tau)\Delta g(t)\rangle_{\rm c}}{\langle \Delta g(t)^2\rangle_{\rm c}} d\tau, \tag{SI D.5}$$

where Γ' is the measurement rate of the mode and $\langle \Delta g(t+\tau)\Delta g(t)\rangle_{\rm c}$ is the correlation function of the atomic motion (introduced in Sec. B). Experimentally, we find that this spectrum at frequencies close to the resonance has a Gaussian shape (consistent with a non-Markovian thermal bath), and describe it using the expression

$$S_{PP,\rm ext}[\Omega]/\mathrm{SN} = A_b\, e^{-(\Omega-\Omega_{\rm S})^2/(2\gamma_b^2)}, \tag{SI D.6}$$

where A_b is the magnitude of the added noise on resonance in shot noise (SN) units, and γ_b is the characteristic decay rate. The spectral width of the broadband noise is closely related to the transition time τ of atoms through the probe beam, $\gamma_{\rm b} \sim 1/\tau = v_{\rm th}/w$, where w is the width of the beam, $v_{\rm th} = \sqrt{2k_{\rm B}T/M_{\rm Cs}} \approx 200\,\mathrm{m/s}$ is the thermal velocity atoms, $T = 52\,^\circ\mathrm{C}$ is the operating temperature, $k_{\rm B}$ is the Boltzmann constant and $M_{\rm Cs}$ is the mass of one atom.

At the detuning of the optical probe equal to 0.7 GHz, at which the measurement rate of the spin reaches the oscillation frequency, the fast-decaying mode of the atomic ensemble is in the backaction-dominated regime. We therefore include it as an extra oscillator in Eqs. (SI D.1—SI D.3). This approach effectively approximates the correlation function of the thermal motion of the mode by an exponential, which in the spectral domain may introduce an error in the frequency window of several hundreds of kHz around the resonance, much smaller than the full bandwidth of the fit (several MHz).

The full comparison between the model and the experimental data at different optical detunings is shown in Fig. SI2. The data obtained at 7 GHz optical detuning is described by the response of a single oscillator mode to the measurement backaction. The data obtained at 3 GHz detuning is described with $n_{\rm modes} = 2$. At 0.7 GHz, we include the fast-decaying mode in the model and describe the experiment with $n_{\rm modes} = 3$. The homodyne spectra at all quadratures are processed in one global fit, where the resonance frequencies Ω_i, the measurement rates Γ_i, the dynamical backaction coefficients ζ_i, the intrinsic damping rates $\gamma_{0,i}$, and the quadrature angles ϕ are free parameters, and the values of the thermal occupancy $n_{\rm th}$ and the detection efficiency η are taken from independent calibrations. When processing the broadband measurements at 0.7 GHz, we additionally correct for the frequency response of the measurement electronic chain. The total quantum cooperativity for the data in Fig. SI2c is 4.6.

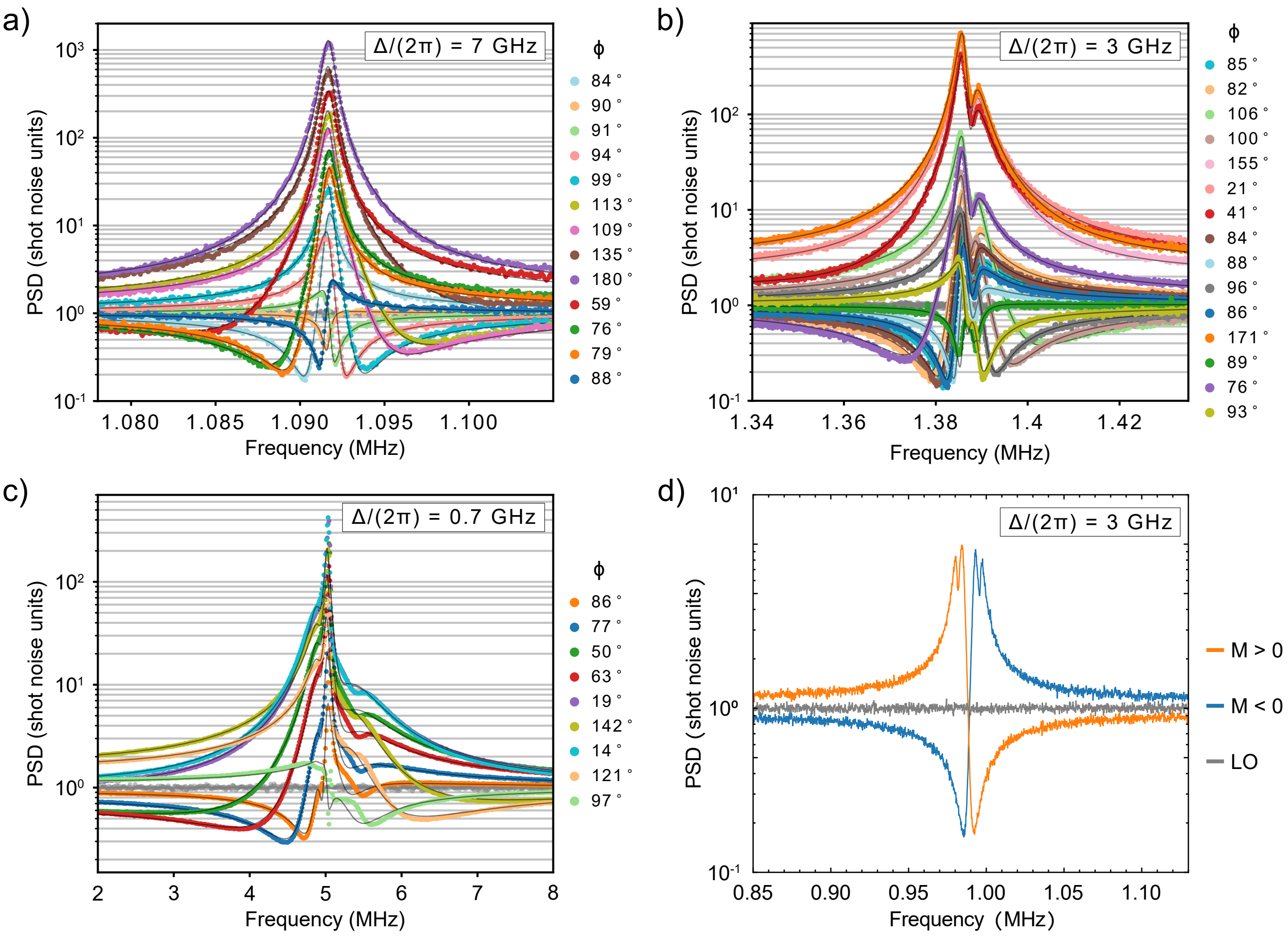

FIG. SI2. a)-c) Power spectral densities (PSD) of homodyne signals recorded at different quadrature angles ϕ and laser detunings Δ. The points of different colors show the experimental spectra for different quadrature angles as labeled in the legends. The black curves show the results of global fits at each detuning performed as described in Sec. D. Gray points show the local oscillator shot noise. Panel a) displays only part of the 17 traces fitted in total. d) The effect of changing the oscillator mass, M, on the homodyne spectrum measured at a quadrature intermediate between $\hat{X}_{\mathrm{L}}$ and $\hat{P}_{\mathrm{L}}$. The blue curve shows the spectrum recorded in a negative mass (M) configuration, the orange curve shows the spectrum recorded in a positive mass configuration, and the gray curve shows the local oscillator (LO) shot noise. The sign of the mass was changed by inverting the direction of the magnetic field with respect to the x axis. The spectra were recorded using a 12 mW probe detuned from the optical transition by 3 GHz.

Appendix E: The sign of the mass

Spin oscillators can have positive or negative effective masses depending on the orientation of the mean spin alignment $\langle \hat{J}_x \rangle$ with respect to the magnetic field. The sign of the mass determines the overall sign of the response $\chi[\Omega]$ of the oscillator to generalized forces, including the quantum backaction force when the oscillator is subjected to linear measurements. Negative-mass oscillators can cancel measurement backaction on regular material oscillators [1], and become entangled with them [5].

The sign of the oscillator mass, together with the detection angle and the Fourier frequency, determines the sign of the backaction-imprecision correlations observed in homodyne measurement records. For multiple resonances, it also inverts the signs of the frequency splittings due to the Stark and quadratic Zeeman effects. The total effect of inverting the mass sign on homodyne spectra is therefore the reflection of the spectra with respect to the Larmor frequency. We observe this in Fig. SI2d, where we invert the sign of the mass by changing the direction of the magnetic field.

Parameter	Symbol	Value	
**Single-oscillator model: 7 GHz detuning**			
Readout rate	$\Gamma/2\pi$	13.2 kHz	
Intrinsic damping rate	$\gamma_0/2\pi$	0.409 kHz	
Thermal decoherence rate	$\gamma_{\rm th}/2\pi$	1.146 kHz	$= (2n_{\rm th}+1)\gamma_0$
Tensor coefficent	ζ	0.012	
Resonant frequency	$\Omega/2\pi$	1.092 MHz	
Detection efficiency	η	91%	(fixed)
Magnitude of fast-decaying modes	A_b	1.02 SN	
Decay rate of fast-decaying modes	$\gamma_b/2\pi$	0.160 MHz	(fixed)
**Two-oscillator model: 3 GHz detuning**			
Readout rates	$\Gamma_1/2\pi$	32.3 kHz	
	$\Gamma_2/2\pi$	19.8 kHz	
Intrinsic damping rates	$\gamma_{1,0}/2\pi$	0.940 kHz	
	$\gamma_{2,0}/2\pi$	1.772 kHz	
Thermal decoherence rates	$\gamma_{1,\rm th}/2\pi$	2.633 kHz	$= (2n_{\rm th}+1)\gamma_{1,0}$
	$\gamma_{2,\rm th}/2\pi$	4.963 kHz	$= (2n_{\rm th}+1)\gamma_{2,0}$
Tensor coefficients	ζ_1	0.022	
	ζ_2	0.050	
Larmor frequencies	$\Omega_1/2\pi$	1.386 MHz	
	$\Omega_2/2\pi$	1.389 MHz	
Detection efficiency	η	91%	(fixed)
Decay rate of fast-decaying modes	A_b	2.20 SN	
Magnitude of fast-decaying modes	$\gamma_b/2\pi$	0.160 MHz	(fixed)
**Three-oscillator model: 0.7 GHz detuning**			
Readout rates	$\Gamma_1/2\pi$	0.639 MHz	
	$\Gamma_2/2\pi$	0.559 MHz	
	$\Gamma_3/2\pi$	0.569 MHz	
Intrinsic damping rates	$\gamma_{1,0}/2\pi$	0.019 MHz	
	$\gamma_{2,0}/2\pi$	0.231 MHz	
	$\gamma_{3,0}/2\pi$	0.176 MHz	
Thermal decoherence rates	$\gamma_{1,\rm th}/2\pi$	0.052 MHz	$= (2n_{\rm th}+1)\gamma_{1,0}$
	$\gamma_{2,\rm th}/2\pi$	0.648 MHz	$= (2n_{\rm th}+1)\gamma_{2,0}$
	$\gamma_{3,\rm th}/2\pi$	0.492 MHz	$= (2n_{\rm th}+1)\gamma_{3,0}$
Tensor coefficients	ζ_1	0.089	
	ζ_2	0.335	
	ζ_3	0.024	
Larmor frequencies	$\Omega_1/2\pi$	4.984 MHz	
	$\Omega_2/2\pi$	5.271 MHz	
	$\Omega_3/2\pi$	4.888 MHz	
Detection efficiency	η	89%	(fixed)

TABLE SI1. The parameters of the theoretical models presented in Fig. SI2 a)-c), including those obtained from the fits (no mark in the right column), those calibrated separately (marked as fixed), and those derived from other parameters (with the formula given in the right column). The thermal occupancy was fixed in all models to the separately calibtated value $n_{\rm th} = 0.9$.

Appendix F: The analytical expressions for the spectra of optical squeezing

a. *The simplest case—a single oscillator with pure position-measurement interaction*

We begin by deriving the spectrum of the detected light given in Eq. 2 of the main text, by solving the equation of motion for a single-mode spin oscillator with $\zeta = 0$, and finding the output signal using the input-output relations. Towards this end, we need to introduce the Fourier transforms of Hermitian operators. For a Heisenberg-picture operator $\hat{Z}(t)$, the Fourier transform is defined as

$$\hat{Z}[\Omega] \equiv \frac{1}{\sqrt{2\pi}} \int_{-\infty}^{\infty} \hat{Z}(t) e^{-i\Omega t} dt, \qquad \text{(SI F.1)}$$

and related to the spectral density of the observable Z as

$$\left\langle \hat{Z}[\Omega]\hat{Z}[\Omega']^{\dagger} \right\rangle = S_Z[\Omega]\, \delta(\Omega - \Omega'). \qquad \text{(SI F.2)}$$

The Fourier domain input-output relations for the light field are given by

$$\hat{P}_{\mathrm{L}}^{\mathrm{out}}[\Omega] = \hat{P}_{\mathrm{L}}^{\mathrm{in}}[\Omega] + \sqrt{\Gamma}\,\hat{X}_{\mathrm{S}}[\Omega], \qquad \hat{X}_{\mathrm{L}}^{\mathrm{out}}[\Omega] = \hat{X}_{\mathrm{L}}^{\mathrm{in}}[\Omega]. \tag{SI F.3}$$

The temporal evolution of the spin is governed by the equation

$$\frac{d^2}{dt^2}\hat{X}_{\mathrm{S}}(t) + \gamma_0 \frac{d}{dt}\hat{X}_{\mathrm{S}}(t) + \Omega_{\mathrm{S}}^2 \hat{X}_{\mathrm{S}}(t) = 2\sqrt{\Gamma}\hat{X}_{\mathrm{L}}^{\mathrm{in}}(t) + \sqrt{2}\hat{F}(t), \tag{SI F.4}$$

where $\hat{F}(t)$ is the thermal force with the correlation $\langle \hat{F}(t_1)\hat{F}(t_2)\rangle = \gamma_0(n_{\mathrm{th}} + 1/2)\delta(t_1 - t_2)$. Based on this equation, we relate the Fourier spectrum of the spin motion to the input noises (comprised of the vacuum fluctuations of light and the intrinsic "thermal" noise) as

$$\hat{X}_{\mathrm{S}}[\Omega] = \chi[\Omega]\left(2\sqrt{\Gamma}\hat{X}_{\mathrm{L}}^{\mathrm{in}}[\Omega] + \sqrt{2}\hat{F}[\Omega]\right), \tag{SI F.5}$$

where $\chi[\Omega] = \Omega_{\mathrm{S}}/(\Omega_{\mathrm{S}}^2 - \Omega^2 - i\Omega\gamma_0)$ is the susceptibility of the spin to generalized forces.

The photocurrent signal detected by homodyning the general quadrature of the output light, $\hat{Q}_{\mathrm{L}}^{\phi}$, which was defined in Eq. (SI C.1), has the following spectrum

$$\left\langle \hat{Q}_{\mathrm{L}}^{\phi}[\Omega]\hat{Q}_{\mathrm{L}}^{\phi}[\Omega']\right\rangle = \frac{1}{4}\delta(\Omega-\Omega') + 2\Gamma\sin(2\phi)\mathrm{Re}\left[\chi[\Omega]\right]\left\langle \hat{X}_{\mathrm{L}}^{\mathrm{in}}[\Omega]\hat{X}_{\mathrm{L}}^{\mathrm{in}}[\Omega']\right\rangle + \Gamma\cos(\phi)^2\left\langle \hat{X}_{\mathrm{S}}[\Omega]\hat{X}_{\mathrm{S}}[\Omega']\right\rangle, \tag{SI F.6}$$

where we used the fact that the only contribution to the spin motion that is correlated with the input light is that proportional to $\hat{X}_{\mathrm{L}}^{\mathrm{in}}[\Omega]$. Finally, by replacing the correlations of the input noises with the known expressions according to

$$\left\langle \hat{X}_{\mathrm{L}}^{\mathrm{in}}[\Omega]\hat{X}_{\mathrm{L}}^{\mathrm{in}}[\Omega']\right\rangle = (1/4)\delta(\Omega-\Omega'), \tag{SI F.7}$$

$$\langle F[\Omega]F[\Omega']\rangle = \gamma_0(n_{\mathrm{th}} + 1/2)\delta(\Omega-\Omega'), \tag{SI F.8}$$

$$\left\langle \hat{X}_{\mathrm{S}}[\Omega]\hat{X}_{\mathrm{S}}[\Omega']\right\rangle = |\chi[\Omega]|^2\left(4\Gamma\left\langle \hat{X}_{\mathrm{L}}^{\mathrm{in}}[\Omega]\hat{X}_{\mathrm{L}}^{\mathrm{in}}[\Omega']\right\rangle + 2\left\langle \hat{F}[\Omega]\hat{F}[\Omega']\right\rangle\right), \tag{SI F.9}$$

we arrive at Eq. 2 of the main text.

b. *Optical squeezing in the presence of dynamical backaction*

To illustrate the effect that the deviation of the interaction Hamiltonian from pure position measurement-type ($\zeta = 0$) has on the detected spectra and the squeezing of light, we present an analytical solution for the optimum-quadrature homodyne spectrum in the single-oscillator model with arbitrary $\zeta \in [-1, 1]$ under the rotating-wave approximation (RWA). Again, for a single mode, by solving Eqs. (SI D.2-SI D.3) and using the input-output relations given by Eq. (SI D.1), we find the spectrum of the output signal neglecting the detection losses as

$$S_{\phi}[\Omega]/\mathrm{SN} = 1 + 2\mathrm{Re}\left[\mathcal{A}\chi[\Omega]\right] + |\mathcal{A}\chi[\Omega]|^2\left(1 + \frac{\gamma_{\mathrm{th}} + \gamma_0}{\Gamma(1+\zeta)}\right), \tag{SI F.10}$$

where $\mathrm{SN} = 1/4$ is the shot noise level, $\chi[\omega] = -(1/2)/(\Delta\Omega + i\gamma/2)$ is the RWA force susceptibility, $\Delta\Omega = \Omega - \Omega_{\mathrm{S}}$ is the Fourier-detuning from the oscillator resonance,

$$\gamma = \gamma_0 + 2\zeta\Gamma, \tag{SI F.11}$$

is the total oscillator linewidth, and the transduction factor $\mathcal{A}$ is

$$\mathcal{A} = i\Gamma(1+\zeta)\left((1+\zeta) + (1-\zeta)e^{-2i\phi}\right). \tag{SI F.12}$$

By minimizing Eq. (SI F.10) over the quadrature angle ϕ, we find the frequency-dependent maximum-squeezing angle ϕ_{min} via

$$\tan\left(2\phi_{\mathrm{min}}[\Omega]\right) = -\frac{2\Delta\Omega}{\gamma_{\mathrm{dec}}}, \tag{SI F.13}$$

where the total decoherence rate $\gamma_{\rm dec} = \gamma_{\rm th} + \gamma_{\rm QBA}$ is the sum of the decoherence rates due to the intrinsic thermal noise, $\gamma_{\rm th}$ and the quantum backaction, $\gamma_{\rm QBA}$ which are defined as

$$\gamma_{\rm th} = (2n_{\rm th} + 1)\gamma_0, \qquad \gamma_{\rm QBA} = \Gamma(1 + \zeta^2). \tag{SI F.14}$$

The shot-noise normalized signal spectrum at the optimum quadrature is

$$S_{\phi_{\min}}[\Omega]/\mathrm{SN} = 1 - \frac{2\gamma_{\rm DBA}/\gamma}{1 + (2\Delta\Omega/\gamma)^2} - \frac{2\gamma_{\rm dec}\Gamma/\gamma^2}{1 + (2\Delta\Omega/\gamma)^2}\left((1-\zeta^2)\sqrt{1 + \left(\frac{2\Delta\Omega}{\gamma_{\rm dec}}\right)^2} - (1+\zeta^2)\right), \tag{SI F.15}$$

where $\gamma_{\rm DBA} = 2\zeta\Gamma$ is the contribution of the dynamical backaction to the total oscillator linewidth (the optical damping). The absolute minimum of the spectrum is found by further minimizing $S_{\phi_{\min}}[\Omega]$ over $\Delta\Omega$, which can be done analytically in the general case, but yields a cumbersome result. Instead of presenting this result, we restrict the attention to the case $\zeta \ll 1$, which is relevant to our experiments, and estimate the minimum noise level by evaluating $S_{\phi_{\min}}[\Omega]$ at $\Delta\Omega_{\min,\zeta=0} = 1/2\sqrt{\gamma(2\gamma_{\rm dec} + \gamma)}$, the optimum Fourier detuning for $\zeta = 0$. The result is

$$S_{\min} \approx 1 - \frac{\Gamma}{\gamma_{\rm dec} + \gamma_0} - \frac{(\gamma_0 + \gamma_{\rm th})\gamma_{\rm DBA}}{(\gamma_0 + \gamma_{\rm dec})^2}. \tag{SI F.16}$$

When the thermal occupancy of the intrinsic bath is close to zero, and the quantum cooperativity is in the intermediate regime, such that $\gamma_{\rm dec}$ has the same order of magnitude as γ_0, there is an improvement in the minimum noise level from a small positive optical damping.

Appendix G: The generation of the collimated tophat beam

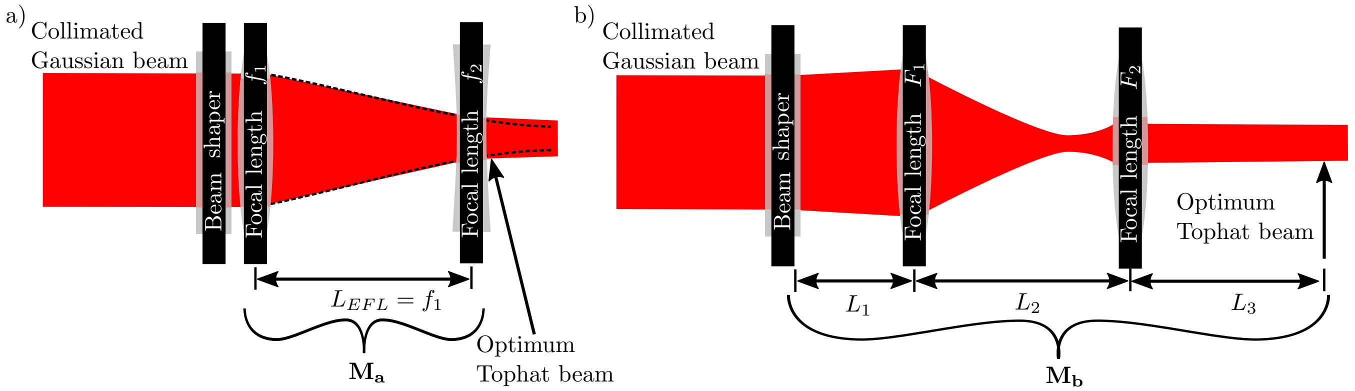

FIG. SI3. Optical setups for the generation of collimated tophat beams. $\mathbf{M}_{a,b}$ are ray transfer matrices. a) A simple setup. The dashed black line shows how the beam would propagate after passing the beam shaper and the lens f_1, but without passing the negative lens f_2. EFL: effective focal length. b) A realistic setup designed using the condition $\mathbf{M}_a = \mathbf{M}_b$. Beam shaper: Gaussian-to-tophat beam-shaping lens.

Optical beams with tophat transverse profiles are commonly produced by passing a collimated Gaussian beam through an aspherical beam shaper, and focusing the beam after the shaper using a spherical lens. In this configuration, the optimum tophat profile (giving the sharpest roll-off of the intensity distribution in the transverse direction) is realized before the focal point, and the beam is tightly focused. In our experiment, it is essential to create a beam in which the tophat profile coincides with the position of the beam waist, and has a relatively large transverse size, enabling a long Rayleigh length extending over the entire cell channel.

An intuition on how to produce a tophat beam that fulfills our criteria can be obtained by examining the setup shown in figure Fig. SI3a, which is a straightforward extension of the usual beam shaper application scheme with an addition of a negative lens f_2. The optimum tophat transverse profile is realized at a distance one effective focal length (EFL) away from the first lens. The transverse width is proportional to the focal length f_1. The beam is converging at the optimum point, because of the full fan angle of the tophat beam shaper (i.e. the divergence the shaper introduces in the beam). By placing an appropriate negative lens f_2 in the optimum point, the beam can be

collimated, and its waist position made coincide with the optimum location of the transverse profile. The required focal length of the negative lens can be calculated given the size of the input Gaussian beam, $w_{\rm in}$, and the full fan angle of the beam shaper, $\phi_{\rm FA}$, as $f_2 = \frac{\phi_{\rm FA}/w_{\rm in} f_1}{\phi_{\rm FA}/w_{\rm in} - 1/f_1}$.

The setup in Fig. SI3a would be challenging to implement directly, because the waist position of the beam is located inside the cell, where placing a lens is hardly realistic. However, one can find an optical setup with an identical ray transfer matrix to the one in Fig. SI3a, but realized using a different physical arrangement of lenses. Such a setup is shown in Fig. SI3b. The transfer matrices for the two setups, $\mathbf{M}_a$ and $\mathbf{M}_b$, are given by

$$\mathbf{M}_a = \mathbf{L}(f_2)\mathbf{S}(f_1)\mathbf{L}(f_1), \qquad \mathbf{M}_b = \mathbf{S}(L_3)\mathbf{L}(F_2)\mathbf{S}(L_2)\mathbf{L}(F_1)\mathbf{S}(L_1), \tag{SI G.1}$$

where the matrices for propagation in free space, $\mathbf{S}$, and passing through a lens, $\mathbf{L}$, respectively, are

$$\mathbf{S}(L) = \begin{bmatrix} 1 & L \\ 0 & 1 \end{bmatrix}, \qquad \mathbf{L}(f) = \begin{bmatrix} 1 & 0 \\ -1/f & 1 \end{bmatrix}. \tag{SI G.2}$$

In our experiment, the setup in Fig. SI3b is implemented using lenses of pre-determined focal lengths F_1 and F_2, while the separating distances L_1, L_2 and L_3 are adjusted to meet the condition $\mathbf{M}_a = \mathbf{M}_b$. Additionally, the matrix $\mathbf{M}_a$ is supplemented by an inversion in the transverse plane, which can be interpreted as passing the beam through an extra 4f optical system, which is done in order to have more flexibility in the choice of lenses and more control over the resulting distances.

* sergey.fedorov@nbi.ku.dk
† polzik@nbi.dk

[1] C. B. Møller *et al.*, Quantum back-action-evading measurement of motion in a negative mass reference frame, Nature **547**, 191 (2017).
[2] B. Julsgaard, J. Sherson, J. L. Sørensen, and E. S. Polzik, Characterizing the spin state of an atomic ensemble using the magneto-optical resonance method, Journal of Optics B: Quantum and Semiclassical Optics **6**, 5 (2003).
[3] R. Shaham, O. Katz, and O. Firstenberg, Quantum dynamics of collective spin states in a thermal gas, Physical Review A **102**, 012822 (2020).
[4] J. Sherson, B. Julsgaard, and E. S. Polzik, Deterministic Atom–Light Quantum Interface, in *Advances In Atomic, Molecular, and Optical Physics*, Vol. 54, edited by P. R. Berman, C. C. Lin, and E. Arimondo (Academic Press, 2007) pp. 81–130.
[5] R. A. Thomas *et al.*, Entanglement between distant macroscopic mechanical and spin systems, Nature Physics , 1 (2020).
[6] D. V. Vasilyev, K. Hammerer, N. Korolev, and A. S. Sørensen, Quantum noise for Faraday light–matter interfaces, Journal of Physics B: Atomic, Molecular and Optical Physics **45**, 124007 (2012).
[7] J. Borregaard *et al.*, Scalable photonic network architecture based on motional averaging in room temperature gas, Nature Communications **7**, 11356 (2016).
[8] C. W. Gardiner and M. J. Collett, Input and output in damped quantum systems: Quantum stochastic differential equations and the master equation, Physical Review A **31**, 3761 (1985).
[9] K. Hammerer, E. S. Polzik, and J. I. Cirac, Teleportation and spin squeezing utilizing multimode entanglement of light with atoms, Physical Review A **72**, 052313 (2005).
[10] V. B. Braginskii and A. B. Manukin, *Measurement of weak forces in physics experiments* (1977) chicago.
[11] R. A. Thomas, *Optical spin-mechanics quantum interface: entanglement and back-action evasion*, Ph.D. thesis, Niels Bohr Institute (2020).
